# Prevalence of Keratosis in the Oral Cavity: A Clinical Retrospective Study

**DOI:** 10.7759/cureus.52199

**Published:** 2024-01-13

**Authors:** Manya Nautiyal, Jayanth Kumar Vadivel, Karthikeyan Ramalingam

**Affiliations:** 1 Oral Medicine and Radiology, Saveetha Dental College and Hospitals, Saveetha Institute of Medical and Technical Sciences, Saveetha University, Chennai, IND; 2 Oral Pathology and Microbiology, Saveetha Dental College and Hospitals, Saveetha Institute of Medical and Technical Sciences, Saveetha University, Chennai, IND

**Keywords:** epidemiology, frictional keratosis, tobacco pouch keratosis, morsicatio buccarum, keratosis

## Abstract

Introduction: A lesion in the oral cavity can appear clinically white due to an increase in keratin (hyperkeratosis), an increase in the thickness of the spongiotic cells (acanthosis), accumulation of fluid within the epithelium, and formation of the pseudomembrane. There are several innocuous white lesions, while only a few require aggressive management. The lesions of frictional keratosis, tobacco pouch keratosis, and morsicatio buccarum (MB) are innocuous white lesions that can be treated with simple treatments. This paper aimed to study the prevalence of the three white lesions among the patients visiting Saveetha Dental College. The objectives were to study the gender prevalence of the lesions and the average age distribution of these lesions.

Methodology: The study was a retrospective clinical study that collected data from DIAS (Digital Information and Archiving Software) over four years among the patients visiting Saveetha Dental College. The inclusion and exclusion criteria were based on the clinical diagnostic features, and the existence of any other oral potentially malignant disorder made the exclusion criteria. The collected data were entered into Statistical Package for the Social Sciences (SPSS) version 26.0 (IBM Corp., Armonk, NY). The data were analyzed for descriptive and inferential statistics.

Results: The total number of patients with the lesions mentioned above was 5,613, with an average age of 37.28 years. The most common lesion was frictional keratosis with 4,026 patients, followed by tobacco pouch keratosis with 1,537 patients. The least common was MB, with 54 patients. Based on gender, all of the above lesions were most commonly seen in men. The age analysis revealed that frictional keratosis and MB were seen in middle age, while tobacco pouch keratosis was seen in middle to old age.

Discussion: From the above study, it is noticed that frictional keratosis was the most common lesion, which had arisen most commonly in the third molar region and was seen in the younger age group due to the eruption of the third molars. Among the lesions, the only tobacco-induced lesion was tobacco pouch keratosis, which was seen predominantly in middle to old age, implying that prolonged habit usage was essential for the lesion to develop.

Conclusion: This study has helped us understand the prevalence of innocuous keratotic lesions in the South Indian population. The findings of this study will give a guideline for confirming the clinical diagnosis of similar lesions. This could potentially reduce the need for undertaking a biopsy and rather try treating with less invasive modalities, and when no response is seen, then it would be worthwhile to do a biopsy.

## Introduction

A lesion in the oral cavity that appears white may be caused by an increase in keratin (hyperkeratosis), an increase in the thickness of spongiotic cells (acanthosis), accumulation of fluid within the epithelium, and the formation of pseudomembrane [1]. Among the histological causes, the most common reason is hyperkeratosis, which is triggered by several benign etiologies and could eventually become malignant if left untreated. Though most white lesions are benign, a few are categorized as oral potentially malignant disorders (OPMD). The most common lesion of OPMD is leukoplakia [1]. Most white lesions arising from keratosis may be due to friction from sharp teeth or tobacco-related habits. The tobacco ingredients, mainly nicotine and nitrosamines, cause an epithelial thickness, which makes the lesion clinically appear white. The initial stage of the lesion is harmless as it causes epithelial hypertrophy [2].

The usage of smokeless tobacco (SLT) products is on the rise in Southeast Asia countries. In India, SLT use is the highest in women, and it dominates as the second most common tobacco usage habit after smoking in males. There is also an increased usage in people of the 3rd and 4th decades because of the addiction to the habit and difficulty in quitting the habit [3].

Using SLT causes white, grey, or pale patches in contact places, such as the buccal mucosa or alveolar mucosa [4]. The nitrosamines in the SLT cause the main epithelial changes and are the commonly implicated carcinogen. One of the variants of SLT is the moist snuff with higher alkalinity and better penetration into the oral mucosa [5]. Moist snuff is associated with a higher prevalence of tobacco pouch keratosis [6]. The prevalence has been as high as 60% with the usage of SLT [7]. The SLT items available in India include betel quid with tobacco, khaini, gutka, pan masala with tobacco, zarda, mishri, mawa, gul, bajjar, and gudakhu, and are extensively used and accessible. These objects can be sucked, eaten, or stuck between the teeth, the gums, or the cheeks [8,9]. Every third adult in rural India and every fifth adult in urban India use tobacco in some way, according to the Global Adult Tobacco Survey-2 (GATS 2). In India, 28.6% (266.8 million) of adults who are 15 years of age or older use tobacco in some way. Men use tobacco at a rate of 42.4%, while women use it at a rate of 14.2% in India [10].

Among the smokers, the most popular tobacco product in India is bidi, which is smoked by 7.7% of adult Indians, and the second most common is khaini, a tobacco and lime mixture that is used by one in every nine persons (11.2%) [9]. In third place is the usage of guthka, which is a concoction of tobacco, lime, and areca nut, followed by the plain consumption of betel quid and tobacco [11]. SLT products are used more frequently as a result of accessibility and affordability, including through illegal trafficking. SLT products are still sold and possessed even in jurisdictions where they are prohibited [9].

Some white lesions are clinically innocuous. One good example is the occurrence of frictional keratosis. This occurs due to trauma from sharp teeth, toothbrushes, or dental appliances. The reported prevalence of frictional keratosis ranges from 0.26% to 5.3% [12]. Sometimes, another innocuous white lesion, like linea alba, is mistaken for a potentially malignant disorder [13]. Morsicatio buccarum (MB) is also a lesion brought on by chronic biting of the cheeks, which is a form of obsessive neurosis [14].

The above lesions are reversible when the causative irritant is removed and do not require medical intervention [15]. Hence, it is imperative to distinguish between the different types of keratotic lesions to identify the treatment needs of the population.

With the above background, we wanted to study the epidemiological prevalence of different types of keratotic white lesions in the oral cavity. The objectives were to investigate the prevalence of various keratotic lesions according to the population's gender.

## Materials and methods

The data for our study were collected from the DIAS (Dental Information Archiving Software) of Saveetha Dental College, Chennai, India. The evidence ranged from the patients treated from June 2019 to June 2023. The Institutional Human Ethical Committee of the institution approved the study vide the ethical committee number IHEC/SDC/UG-2157/23/OMED/303. The inclusion and exclusion criteria are mentioned in Table 1.

**Table 1:** Inclusion and exclusion criteria of the samples used in the study. SLT: smokeless tobacco; OPMDs: oral potentially malignant disorders.

Lesion	Inclusion criteria	Exclusion criteria
Frictional keratosis	Clinically identifiable source of irritation or trauma in relation to the lesional site.	If clinically no response to the removal of the irritant after four weeks.
	Clinical appearance of white at the site of contact.	If there are other OPMDs associated.
Tobacco pouch keratosis	History of quid/SLT use.	If no response to anti-oxidants after cessation of the habit after four weeks.
	Clinical appearance of a white velvety patch at the site of contact.	If there are other OPMDs associated with tobacco pouch keratosis.
Morsicatio buccarum	History of cheek biting present.	If there are other OPMDs associated with morsicatio buccarum.

The diagnosis was confirmed based on the specific treatment of the lesions. The obtained data were systematically arranged in an Excel sheet (Microsoft Corp., Redmond, WA), with standard comparing values for evaluation as gender, age, and type of keratosis seen in the patient. For further evaluation, we used the SPSS software (IBM Corp., Armonk, NY) to derive the graphical representation of the results along with a chi-square test estimation.

Data analysis for descriptive and bivariate statistics (chi-square test) is conducted by SPSS version 26.0. The software was used for data entry, graphing, and for statistics. Basic descriptive statistics were provided, and inferential statistics were used to test the hypothesis.

The data were checked for normality, and if found following normality, the chi-square test was used. The internal validity was established since the cases were from an institutional database where the patients were followed up and their response to the treatment helped us confirm the diagnosis. The external validity was verified, where standard protocols were used to record the age (from approved identity cards), and the lesions were assessed by two expert clinicians, which ensured that study findings could be generalized to other population groups.

## Results

The above study screened the entire patient database from June 2019 to May 2023, which yielded 144,056 patients visiting the Oral Medicine Department of Saveetha Dental College. Thus, as it was a unicentric study, only a single population ethnicity was studied (Table 2).

**Table 2:** The mean age and number of cases according to the lesion and the average age.

Year	Frictional keratosis		Tobacco pouch keratosis		Morsicatio buccarum	
	Average age	No. of cases	Average age	No. of cases	Average age	No. of cases
2019(June - December)	25.23	403	45.67	125	28.56	5
2020	26.76	1012	44.08	324	29.34	19
2021	25.94	1170	43.78	491	28.99	18
2022	27.05	986	44.92	401	29.06	6
2023 (January - May)	26.04	455	45.34	196	29.13	6

**Figure 1:**
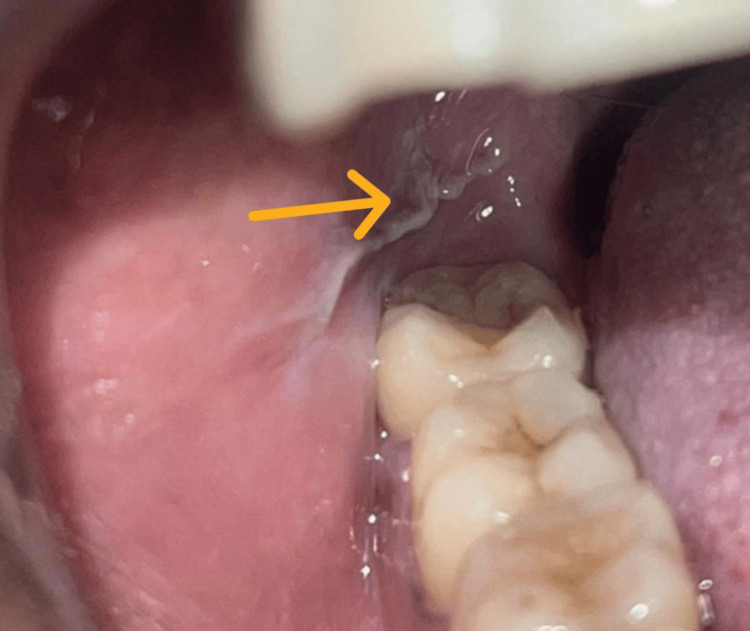
Frictional keratosis presenting as a corrugated white plaque in relation to a sharp tooth from the upper third molar of the right buccal mucosa. The lesion is marked by the orange arrow.

Patients who had presented with frictional keratosis, tobacco pouch keratosis, and cheek biting or MB were identified from this group. Frictional keratosis was identified as a non-scrapable white patch in relation to a sharp tooth or irritant. The following figure shows the presence of a frictional keratosis in relation to a buccoverted 18 (Figure 1).

**Figure 2:**
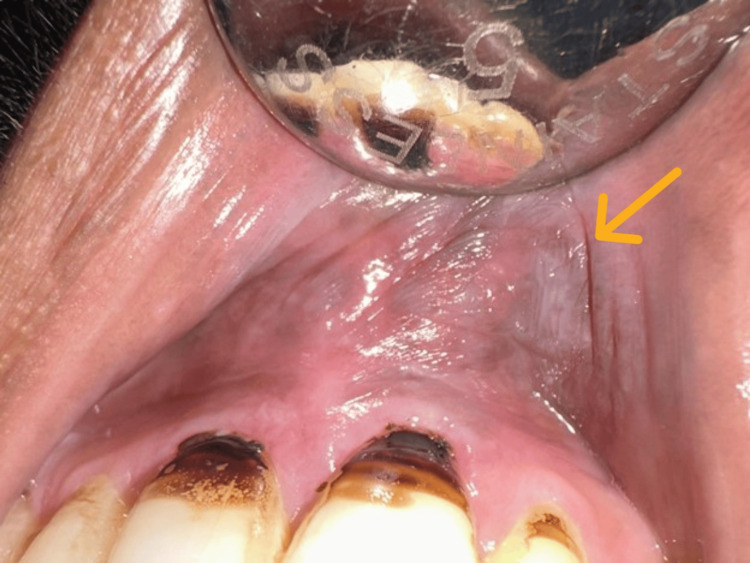
Tobacco pouch keratosis presenting as a white non-scrapable velvety patch in the upper labial vestibule. The lesion is pointed by the orange arrow.

Tobacco pouch keratosis was identified by the presence of a white patch in relation to the place of contact of the SLT product. The clinical photograph below shows the presence of tobacco pouch keratosis in relation to the labial mucosa (Figure 2).

**Figure 3:**
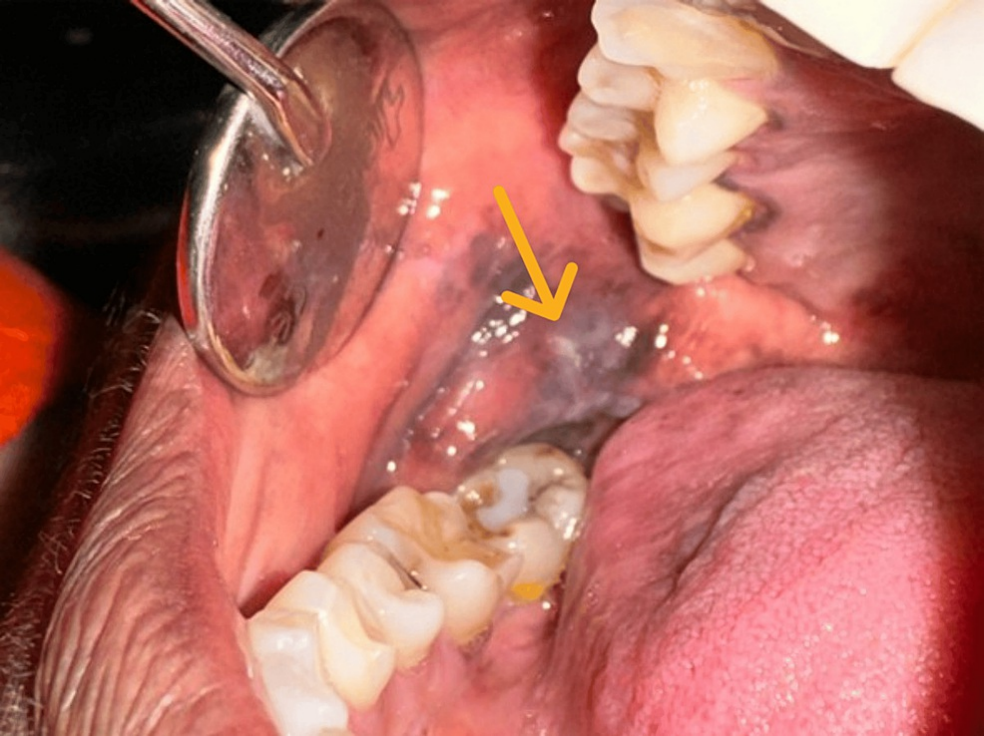
Morsicatio buccarum presenting as greyish white linear patch along the occlusal plane in the right buccal mucosa with hyperpigmentation. The lesion is indicated by the orange arrow.

Cases of MB were clinically identified based on the presence of a white line along the bite plane of the dentition. The clinical photograph shows the presence of MB along the occlusal plane in relation to the posterior region of the buccal mucosa (Figure 3).

The cases were confirmed based on the clinical diagnosis. For frictional keratosis, an identifiable irritation about the lesions was identified. In addition to the clinical findings, the habit of placement of the quid/SLT at the lesion site was verified for tobacco pouch keratosis. For the diagnosis of MB, the presence of a habit of cheek biting at the site of the lesion was considered.

**Figure 4:**
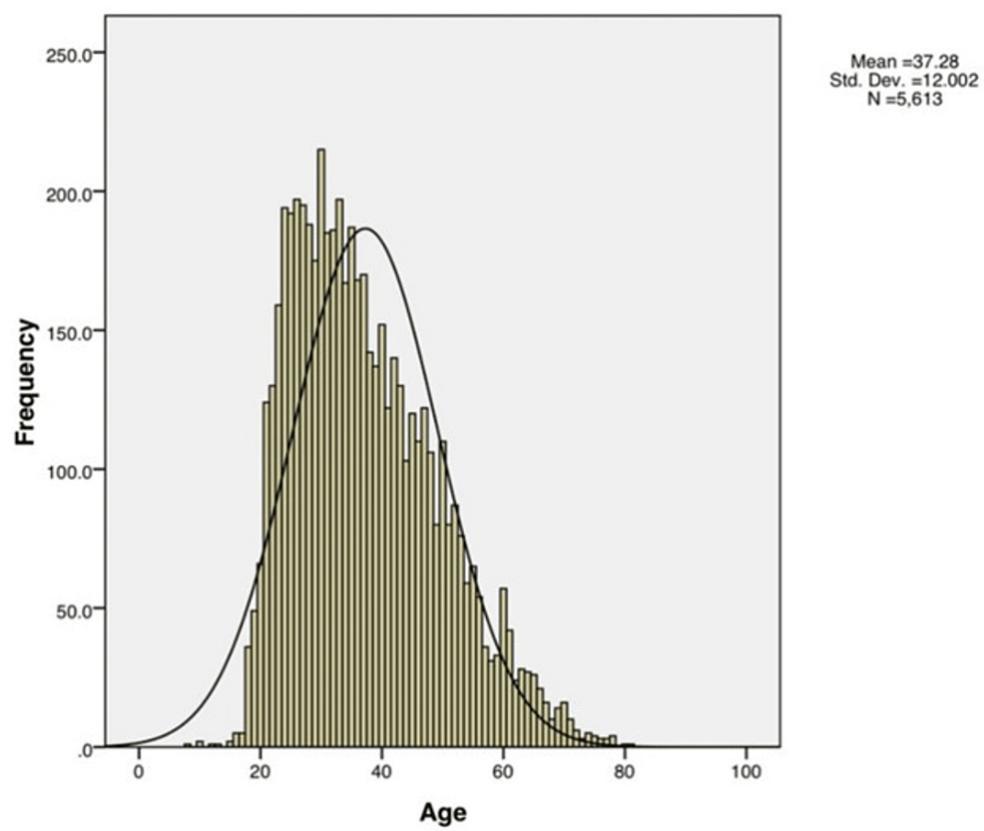
Age distribution of the samples via histogram with bell curve superimposition.

The study had 5,613 patients presenting with one of the above three lesions. This accounts for a prevalence of 3.89% among the patients visiting the dental Institution. The age range of the patients was between 18 and 78 years, with a mean age of 37.28 years. A histogram was drawn considering the ages of the samples, and a standard bell curve was fitted to it, which gave a close approximation. Hence, the data were found to have a normal distribution (Figure 4).

In addition to this, the normality of the individuals' ages was checked using Shapiro-Wilk’s test, which showed that the data had a normal distribution.

**Figure 5:**
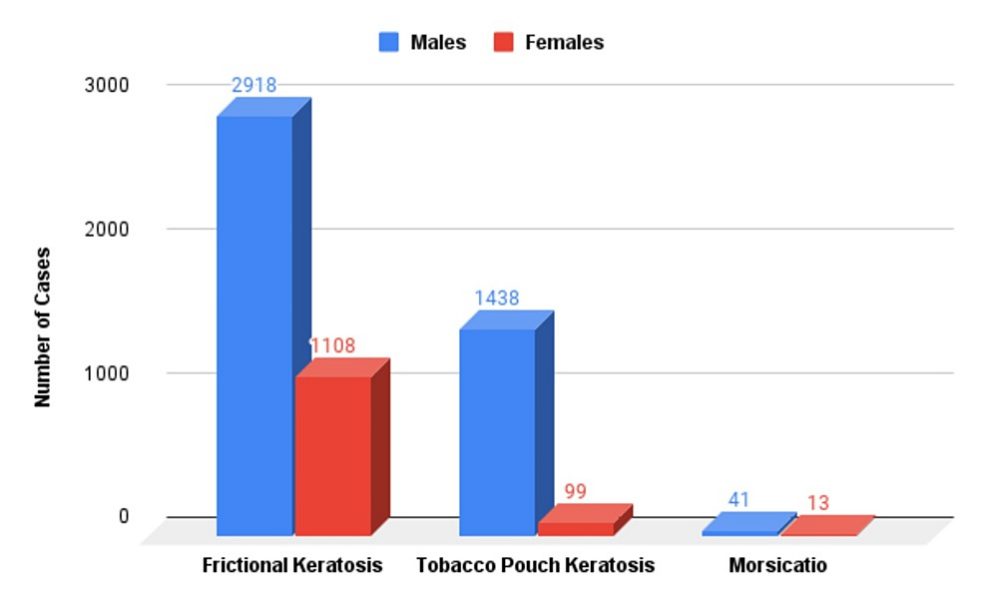
Graphical representation of the prevalence of keratosis and gender distribution.

On a further breakup of the samples based on the age group, it was found that the average age of the patients with frictional keratosis was 26.28, for MB was 29.34, and for tobacco pouch keratosis was 44.53. The bar graph representation of the different types of lesions according to the genders is given in Figure 5.

There were a total of 4022 (71.65%) of patients with frictional keratosis. These data, when split according to gender, had 27.5% of cases in females and 72.5% of cases in males. There were 1537 (27.38%) of the patients with tobacco pouch keratosis. A gender-wise distribution revealed 93.55% of the patients were males, and 6.45% were females. A total of 54 (0.9%) of the patients had MB. A gender-wise distribution of this group yielded 75.92% of the patients were males, and 24.08% were females (Table 3).

**Table 3:** Gender-wise distribution of the samples.

Year	Frictional keratosis		Tobacco pouch keratosis		Morsicatio buccarum	
	Male	Female	Male	Female	Male	Female
2019 (June - December)	219	184	113	12	4	1
2020	634	378	300	24	15	4
2021	863	307	463	23	13	5
2022	868	118	380	21	4	2
2023 (January - May)	334	121	177	19	5	1

Inferential statistics

As the data followed a normal distribution, we tried to see the association between gender and each of the innocuous white lesions. For this purpose, we used a chi-square test with a post hoc test to compare the gender numbers within each lesion category, and it yielded a p-value of 0.01, which was statistically significant. Another post hoc linear by linear association between the gender and the clinical lesion types was also carried out, producing a p-value of 0.034, making it statistically significant.

## Discussion

The above study was conducted to understand better the various types of keratosis, which are clinically innocuous. There is a general phobia among patients and doctors alike that all white lesions of the oral cavity have a potentially malignant disorder. Through this clinical study, we could establish that 71.62% of lesions noted in the study belonged to the group of frictional keratosis. This is in line with the study conducted by Müller et al. [1]. If there can be a known identifiable source of irritation or parafunctional habit, we can always consider eliminating the trauma before subjecting the patient to a biopsy. Patient information is a crucial aspect of managing and treating keratosis. Since frictional keratosis is entirely benign, no aggressive treatment is necessary, and just removal of the etiology is needed. However, it should be halted if the patient generates the lesion due to a habit.

A literature survey was done to study the etiology and epidemiology of each lesion. This was done to study the disease prevalence in the population. Though the etiology of frictional keratosis is well established, the true prevalence of the lesion is unknown, as most of the lesions have been misdiagnosed as leukoplakia [12]. A study done among children by Pinto et al. found that the prevalence of frictional keratosis was 0.26-5.3% [11]. In our study, the vast majority of cases recorded were frictional keratosis, and the age group predominant was in the 3rd decade, with the average age being 26.23 years. These frictional keratosis present as well-defined white patches that are nonscrapable. In rare instances, these lesions may have a corrugated appearance and can have areas of ulceration mixed with them [12]. There have also been cases of linea alba being mistaken as frictional keratosis. However, linea alba is a condition triggered by limited buccal overjet and not a point irritation, as in the case of frictional keratosis [13]. The most common sites for the occurrence of tobacco pouch keratosis are the buccal mucosa and retromolar pad area, which was also noticed in our study, and the following most everyday site is over the crest of the edentulous alveolar ridges [12].

The following typical lesion noted in our study was tobacco pouch keratosis. This lesion is triggered by constant frictional irritation of the rough and abrasive components found in the betel quid rather than a direct effect of the quid components in the mucosa. The severity of the lesion depends on the type of quid used, habit frequency, and the duration of contact of the quid on the mucosa [16]. The constant irritation from the quid can cause a deposition of fibrin-like material in the submucosal, which undergoes secondary keratinization of the overlying epithelium. Clinically, these lesions present as a white corrugated plaque with areas of erythema and are painless. When the lesion is stretched, distinct folds or pockets are noticed [1]. This lesion typically resolves in five to six weeks after the cessation of the habits [16]. In the Indian subcontinent, this tobacco pouch keratosis has been reported as the most common lesion among SLT users. The most common age group reported is the 4th to 5th decade of life, and in our study, the average age of tobacco pouch keratosis patients was 44.53 years [8].

The next lesion studied in our study was MB. This lesion is a continuation of the linea alba, which results from chronic cheek biting, causing keratinization along the buccal mucosa. It occurs as a result of sustained mild trauma and does not carry any carcinogenic risk. However, apart from reduced buccal overjet, the lesion may also be triggered due to anxiety, which results in the patient consciously or unconsciously biting their cheeks and also results from psychiatric disorders, learning disabilities, and rare syndromes like Lesch-Nyhan syndrome and Riley-Day syndrome [17]. The average age of the lesion being reported is in the 3rd decade, and our study also said that the average age was 29.34 years.

Our study concluded that tobacco pouch keratosis was more commonly seen in men. However, a survey by Naveen Kumar et al. stated otherwise [18] that women were found to be practicing tobacco consumption habits more than men. We also observed that frictional keratosis was more commonly found in men than women. A study by Arora et al. [19] concluded that females were found to have frictional keratosis more commonly than men as they are more prone to microtrauma induced due to several household activities [20]. Another study by Majorana et al. stated that in a 10-year experience wherein the patients were aged 0-12 years, MB was more commonly found in females, although there was a slight difference [20]. This study was inconsistent with ours, as we noticed MB is a more common finding in males than females.

Limitations

Though the study was hospital-based, highlighting the different types of white lesions, the study was unicentric. Though the patients were followed up in the institution, and the diagnosis was confirmed based on the treatment response, including a histopathological investigation would have added more strength to the diagnosis. There could be future studies analyzing the different treatment options and the rapidity of cure of the lesions with patient-specific measures.

## Conclusions

We conclude that our study showed the prevalence of frictional keratosis, tobacco pouch keratosis, and MB in the patients of a four-year retrospective follow-up analysis. The paper analyzed the epidemiologic prevalence of the different types of lesions in the patients. In our study, it was found that frictional keratosis was the most commonly seen innocuous keratotic lesion. All the lesions were more commonly seen in men than in women. Since the diagnosis of all the cases in this study was based on clinical diagnosis and a favorable response to therapy, this study could help the clinician identify the lesion. This could also mean that biopsy can be deferred and conducted when there is no response to the therapy. From this study, we can conclude that all white lesions cannot be considered potentially malignant, and there are several innocuous white lesions as well.
